# Downregulation of VRK1 Inhibits Progression of Lung Squamous Cell Carcinoma through DNA Damage

**DOI:** 10.1155/2023/4533504

**Published:** 2023-07-28

**Authors:** Ning Du, Boxiang Zhang, Yunfeng Zhang

**Affiliations:** Department of Thoracic Surgery, The First Affiliated Hospital of Xi'an Jiaotong University, 277 West Yanta Road, Xi'an 710061, Shaanxi, China

## Abstract

**Background:**

Lung squamous cell carcinoma (LUSC) is a common malignancy. And the antitumor effect of bovine pox virus-associated kinase 1 (VRK1) is becoming a hot research topic.

**Methods:**

VRK1 expression and prognosis in LUSC were analyzed using the GEPIA database. The expression of VRK1 mRNA was detected in 25 LUSC clinical tissue samples by RT-PCR. VRK1 shRNA was transfected into LUSC NCI-H520 and SK-MES-1 cell lines to interfere with VRK1 expression, and the efficiency of VRK1 shRNA interference was detected by the western blot. The effects of VRK1 downregulation on LUSC cell viability, migration, cell cycle, and apoptosis were analyzed by the CCK8 assay, scratch assay, transwell assay, and flow cytometry. The effect of VRK1 downregulation on DNA damage response (DDR) was examined by immunofluorescence staining and western blot assays and further validated by in vivo experiments.

**Results:**

VRK1 was highly expressed in both LUSC tissues and cells. Survival analysis showed that the overall survival of LUSC patients with high VRK1 expression was significantly lower than that of LUSC patients with low VRK1 expression (*P*=0.0026). The expression level of the VRK1 gene was significantly higher in cancer tissues of LUSC patients than in paracancerous tissues. After transfection of VRK1 shRNA in both LUSC cells, cell activity decreased (*P* < 0.001), migration ability started to be inhibited (*P* < 0.001), the ratio of G0/G1 phase cells increased (*P* < 0.001), and apoptosis rate increased (*P* < 0.001). Immunofluorescence and western blot results showed that shVRK1 increased the level of *γ*-H2A.X (*P* < 0.001) and promoted apoptosis of tumor cells (*P* < 0.001). In addition, the results of animal experiments showed that shVRK1 had antitumor effects (*P* < 0.001) and a combined effect with DOX (*P* < 0.001).

**Conclusion:**

The downregulation of VRK1 significantly affected the proliferation, apoptosis, migration, and cell cycle progression of LUSC cells via DDR, suggesting that VRK1 is a suitable target for potential LUSC therapy.

## 1. Introduction

Lung cancer is the most prevalent and deadly cancer in the world [1]. The 5-year survival rate was 19.0%, with lung squamous cell carcinoma (LUSC) being a common subtype of non-small cell lung cancer, accounting for 25%–30% of all lung cancers [2]. LUSC is strongly associated with smoking and the most diagnosed cases are in men [3]. Most originate in the larger bronchi and are often central lung cancers with a faster growth rate. The frequency of targeted driver genes in LUSC cells is low and highly heterogeneous, and there are few effective targeted drugs, poor chemotherapy, limited treatment options, and poor prognosis [4], posing great difficulties for precise clinical treatment. Virus-associated kinase 1 (VRK1) is a member of the mitosis-related kinase family in mammals and can be involved in a variety of cellular physiological activities through phosphorylation modifications [5]. Numerous studies [6–8] have demonstrated that VRK1 is differentially expressed in different tumors and has a regulatory role in tumor development. For example, VRK1 is mutated or differentially expressed in glioma, breast cancer, and hepatocellular carcinoma and it regulates the progression of tumor development. The GEPIA database showed VRK1 as the most differential survival gene in LUSC; however, there is no in-depth report on the function of VRK1 in LUSC and its mechanism of action. DNA, as the main component of chromosomes and the main genetic material, is the basis for maintaining the normal physiological functions of cells. In the life process, various endogenous and exogenous factors can cause DNA damage response (DDR), and cells initiate repair or induce apoptosis after DDR. The DDR response pathway is a complex signaling pathway, including DDR repair, apoptosis, and cell cycle regulation. In front of the situation that tumor therapies cannot meet the status quo, researchers have started to explore the possible links and mechanisms between DDR and antitumor drugs, such as those for hepatocellular carcinoma [9] and brain tumor [10], and DDR response pathways have gradually become new antitumor drug targets. However, more research is needed to correlate DDR with LUSC.

In conclusion, this study examines whether and how the downward adjustment of VRK1 has affected the development of LUSC, providing new ideas and a theoretical basis for future clinical diagnosis, prognosis determination, and molecular targeting therapy of LUSC based on VRK1.

## 2. Materials and Methods

### 2.1. Acquisition and Organization of the Database

The GEPIA-LUSC dataset was obtained from the official GEPIA website (https://gepia.cancer-pku.cn/). The dataset included genetic sequencing data and prognostic data of 486 patients with LUSC tissue and 338 patients with adjacent normal lung tissue for survival analysis.

### 2.2. Immunohistochemistry (IHC)

IHC data were downloaded from Protein Atlas (https://www.proteinatlas.org). IHC assessments were conducted using Anti-VRK1 (# 28018-1-AP, Proteintech) based on staining intensity (0–3) and extent (0–4). VRK1 protein expression was divided into two groups: high (score: 4–7) and low (score: 0–3). Five fields of view of each IHC slide were randomly selected for evaluation, and all IHCs were independently and blindly assessed and scored by the investigator (scale bar is 200 *μ*m).

### 2.3. VRK1 Gene Expression Assay in Cancerous and Paraneoplastic Tissues of LUSC Patients

Twenty-five LUSC patients admitted to the First Affiliated Hospital of Xi'an Jiaotong University from March 8, 2021, to March 8, 2022, were selected for the study, and the postoperative LUSC tissues and paraneoplastic tissues were collected and pathologically confirmed. None of the patients received radiotherapy, chemotherapy, interventional therapy, or targeted therapy before surgery; all subjects signed an informed consent form; and the study was approved by the Ethics Committee of the First Affiliated Hospital of Xi'an Jiaotong University. The patient information is shown in Table 1.

### 2.4. Quantitative Real-Time RT-PCR (qRT-PCR)

The supernatant was added to chloroform at a ratio of trizol: chloroform = 5 : 1, mixed well, left to stand for 10 min, stratified, and centrifuged at 4°C for 15 min at 12,000g. The aqueous phase was added to an equal volume of isopropanol, shaken and mixed well, and left to stand for 10 min at room temperature. At 4°C, the samples were centrifuged at 12,000 g for 10 min, and the supernatant was discarded. Subsequently, 75% ethanol was added, followed by centrifugation at 7500 g for 5 min at 4°C, and then the supernatant was discarded. Let the RNA precipitate dry at room temperature for 5–15 min, add an appropriate amount of DEPC-treated water to dissolve the precipitate, and store it in the refrigerator at −20°C. And cDNA was synthesized by reverse transcription using the RNA cDNA First Strand Synthesis Kit (All-Formula Gold-AT341). qRT-PCR was performed using Sybr Green qPCR Master Mix (Biotech-B639273). The internal reference was glyceraldehyde-3-phosphate dehydrogenase (GAPDH).

### 2.5. Western Blotting Experiment

After transfection was completed for 24 h, each group of NCI-H520 and SK-MES-1 cell lines was collected, rinsed 3 times with cooled PBS buffer, and 150 *μ*l of Lysis buffer (Beyoncé) was added to each well. The cell homogenates were collected and crushed using an ultrasonic crusher and centrifuged at 4°C for 10 min at 13000 r/min, and total intracellular proteins were extracted. Protein concentration was determined by the BCA Protein Analysis Kit (Beyoncé). After the gel preparation, 40 *μ*g of each well was sampled for immunoelectrophoresis, and after the transfer membrane was completed, the PVDF membrane was washed with TBS to remove the transfer buffer. Then, it was incubated in 3% BSA on a shaker and closed at room temperature for 1 h, and incubated primary antibody: anti-VRK1 (# 28018-1-AP, Proteintech); anti-Bax (#50599-2-Ig, Proteintech); anti-Bcl2 (#68103-1-Ig, Proteintech); Anti-H2AX (#10856-1-AP, Proteintech); anti-caspase (#319677-1-AP, Proteintech); and anti-GAPDH (#60004-1-Ig, Proteintech). Dilute the primary antibody with 3% BSA containing the preservative NaN_3_ (internal reference at a ratio of 1 : 2000; other proteins are generally diluted at a ratio of 1 : 1000), incubate on a shaker at 4°C overnight; wash the membrane; and reuse the primary antibody recovery. After incubation, the PVDF membrane was washed 3 times with 1 × TBST for 10 min each time, and all protein bands were observed in the ECL color development system.

### 2.6. Cell Lines

Human lung squamous cell carcinoma cells (NCI-H520 and SK-MES-1) were purchased from Shanghai Zhongqiao Xinzhou Biotechnology Co., Ltd; human normal lung epithelial cells (BEAS-2B) were purchased from Shanghai Wing and Applied Biotechnology Co. Both cells were identified by STR before the experiment, and mycoplasma detection was performed by the mycoplasma detection kit.

### 2.7. Cell Transfection

The shRNA sequences targeting VRK1 were constructed by Guangzhou RiboBio Co., Ltd., and the efficacy of transfection was assessed using western blot analyses. The VRK1 shRNA sequence was 5′-GCAGCUAAGCUUAAGAAUUTT-3′. The negative control shRNA (shNC) sequence was 5′-UUCUCCGAACGUGUCACGUTT-3′. VRK1 was downregulated by shRNA in NCI-H520 cells. The riboFECT™ CP Buffer (10x) (Guangzhou RiboBio Co. Ltd.) was prepared by diluting the riboFECT™ CP Buffer (1x) with PBS. After removing the riboFECT™ CP reagent (Guangzhou RiboBio Co., Ltd.), it was shaken thoroughly in a vortex shaker and then left at room temperature to recover to room temperature before use. Cells underwent transfection when in the logarithmic growth phase. NCI-H520 cells were cultured in 6-well plates at a density of 4 × 10^5^ cells/well at 37°C in the presence of 5% CO_2_. The transfection was performed at 37°C for 24 h when the confluency of the cells was ∼30–50%. The concentration of shRNAs transfected was 50 nM. Subsequent experimentation was performed 24 h after transfection.

### 2.8. CCK-8 Assay

After transfection for 48 h, shVRK1 NCI-H520/SK-MES-1 and shNC NCI-H520/SK-MES-1 cells were inoculated in 96-well plates, and six replicates were set up for each group. 10 *μ*L of CCK-8 reagent (Tojin, Japan) was added at 0 h, 24 h, 48 h, 72 h, and 96 h, respectively. The absorbance values of each well were measured at 450 nm on an enzyme marker.

### 2.9. Cell Cycle

The cell culture medium was collected into 15 ml centrifuge tubes before use. After trypsin digestion of the cells, the medium was added to a 15 ml centrifuge tube, and the supernatant was discarded by centrifugation at 1000×g for 5 min. Cells were resuspended in 1 ml of prechilled PBS, placed in a 1.5 ml centrifuge tube, and centrifuged at 1000×g for 5 min to discard the supernatant. The cells were then added to 1 ml of prechilled 70% ethanol, blown and mixed, fixed at 24°C for 24 h, and centrifuged at approximately 1000×g for 5 min as a drop. After the supernatant was discarded, the cells were resuspended in 1 ml of prechilled PBS. Subsequently, 0.5 ml of PI staining solution was added to each tube to resuspend the cell precipitate, which was placed in a 37°C water bath for 30 min and stored at 4°C away from light. Wash off the fixative with PBS before staining. Centrifugal washing conditions: 1000 rpm, 3 min. Add 500 *μ*L PI/RNaseA staining working solution prepared in advance, at room temperature, and protected from light for 30−60 min. Flow cytometry (Beckman Coulter) was completed within 24 h after staining. A red fluorescence was detected at an excitation wavelength of 488 nm. This experiment was performed to analyze the spectrum of cell cycle features after identifying the cells. Applying a gate, the signal of FxCycle Violet was chosen proportional to the amount of DNA throughout the spectrum, which has two typical Gaussian forms, the second with an average value equal to twice the first peak and a plateau in the middle. These three distributions represent cells in the G1 phase, G2/M phase, and S phase, respectively. This experiment was performed on 100,000 cells, removing cellular debris and dead cells, with a final inclusion of 95%. The distribution of the cell cycle was determined using Flow Jo. The experiment was repeated three times, and the results were averaged.

### 2.10. Transwell Assay

After the transfection was completed and when the cell densities of the experimental group of NCI-H520 and SK-MES-1 cell lines and the negative control group reached about 90%, they were spread into 24-well plates. For cell counting, cells were washed and suspended using serum-free medium Opti-MEM to achieve a cell density of 1 × 10^4^ cells per ml, and 300 *μ*l DMEM medium was added to each chamber (i.e., 3000 cells). The lower chamber was added with 600 *μ*l of DMEM medium containing serum and placed in the incubator for 12 h. Cell migration was detected. The cells in the upper chamber were wiped off using a cotton swab, and the cells in the small chamber were soaked in a crystal violet staining solution. The cells were ready to be observed after staining. Three fields of view were randomly selected under the microscope, the number of cell migrations in the small chamber was counted, and the average value was taken for statistical analysis.

### 2.11. Wound Healing Assay

After transfection for 48 h, shVRK1 NCI-H520/SK-MES-1 and shNC NCI-H520/SK-MES-1 cells were grown in six-well plates, respectively, and three replicates were set up for each group. When the cells were opposed to the wall, they were scored with a gun tip. Samples were taken at 0 and 24 hours and photographed.

### 2.12. Apoptosis Assay

Cells were treated with drug administration for 24 h. Cells were washed twice with cold PBS and then resuspended in 1X binding buffer at a concentration of 1 × 10^6^ cells/ml. 100 *μ*l of the solution was transferred (1 × 10^5^ cells) to a 5 ml culture tube. 5 *μ*l FITC AnnexinV and 5 *μ*l PI were added. The cells were gently vortexed and incubated for 15 minutes in the dark at room temperature (25°C). 400 *μ*l of 1X binding buffer was added to each tube. An analysis was performed by flow cytometry (Beckman Coulter) within 1 hour.

### 2.13. Immunofluorescence Experiment

A certain number of cells (about 5 × 10^5^) were inoculated in a six-well plate with preplaced treated coverslips according to the cell situation. After the cells were walled and treated accordingly, the cell plate was removed, the supernatant was discarded, and the cells were washed twice with 1 × PBS. 4% paraformaldehyde was added to completely cover the cells, it was left at room temperature for 15 min, then the paraformaldehyde was removed and washed three times with PBS for 5 min each time. 200 *μ*l of closure solution (4% BSA, 10% secondary antibody source serum, and 0.2% triton 100 diluted with PBS) was added to each well and left to close at room temperature for 1 h. After the closure was completed, 200 *μ*l of primary antibody diluted with closure solution anti-H2AX (#10856-1-AP, Proteintech) (1 : 500) was added to each well and incubated at 4°C. 200 *μ*l of fluorescent secondary antibody was added (1 : 200) and diluted with the blocking solution, incubated for 1 h at room temperature and light was avoided, and it was washed three times with PBS (avoid light) for 5 min each time. Blocker dropwise was added, and then a slide was reverse-clamp and observed by fluorescence microscopy (ThermoFisher Scientific, EVOS M7000).

### 2.14. Animals

Four-week-old male mice, SPF class, weighing about 100 g, were purchased from Changzhou Cavins Laboratory Animal Co. Mice were free ranging in cages and fed normally at 25 ± 2°C. Resuscitated LUSC cells were cultured to the logarithmic growth phase. The cells were digested by trypsin, counted, and resuspended in saline. Nude mice were inoculated with cells subcutaneously and pushed slowly (100–150 *μ*L). Tumor size was measured once a week, and vernier calipers were used to measure the longest and shortest parts of the tumor. *v* = 1/2 *∗* *a* *∗* *b*2 (*a* is the long axis and *b* is the short axis).

### 2.15. Statistical Treatment

SPSS 22.0 statistical software was used to analyze the data. The *t*-test was used to compare the means between the two groups for the measurement data. ROC curves were plotted to analyze the diagnostic efficacy of the VRK1 gene in the GEPIA-LUSC dataset. The VRK1 gene and clinically significant indicators in the GEPIA-LUSC dataset were used to construct a column line graph model, and a survival analysis-based algorithm was used to construct an overall survival assessment model for LUSC. The Kaplan–Meier method and log-rank test were used to analyze and compare the overall survival rates of LUSC patients with different VRK1 expression levels. The difference was considered statistically significant at *P* < 0.05. ^*∗*^*P* < 0.05, ^*∗∗*^*P* < 0.01, and ^*∗∗∗*^*P* < 0.001.

## 3. Results

### 3.1. VRK1 Expression and Prognosis in Lung Cancer Tissues

**Figure 1:**
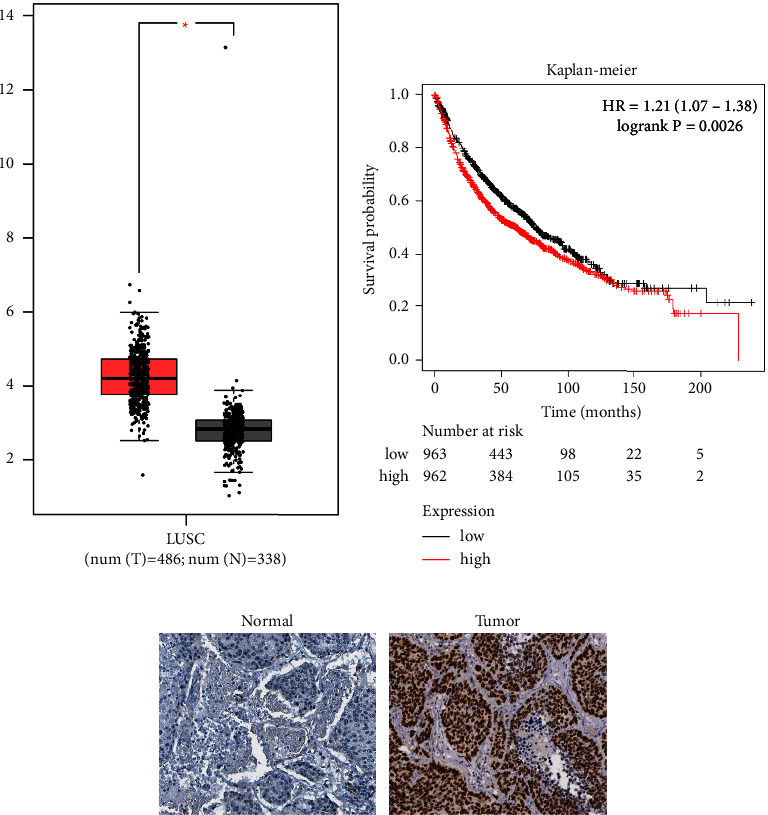
(a) Expression levels of VRK1 in 486 lung cancers and 338 paraneoplastic tissues based on GEPIA-LUSC dataset; (b) Kaplan–Meier survival curves of LUSC patients based on GEPIA-LUSC dataset; and (c) immunohistochemical staining showing VRK1 protein expression in normal and LUSC tissues (200x).

To evaluate the role of VRK1 in LUSC, we first analyzed VRK1 expression in lung cancer tissues and paraneoplastic tissues using the GEPIA database (num (*T*) = 486; num (*N*) = 338). The expression level of VRK1 in lung cancer tissues was significantly higher than that in paraneoplastic tissues (*P* < 0.05) (Figure 1(a)). In addition, we performed a survival analysis of LUSC patients with different VRK1 expression levels using the GEPIA dataset and found that the overall survival rates of LUSC patients with high VRK1 expression were all significantly lower than those of LUSC patients with low VRK1 expression (*P*=0.0026) (Figure 1(b)). Immunohistochemical staining showed a significant increase in the density and intensity of VRK1 expression in lung cancer tumor tissues compared with paraneoplastic tissues (Figure 1(c)).

### 3.2. VRK1 Gene Expression in Cancerous and Normal Tissues

**Figure 2:**
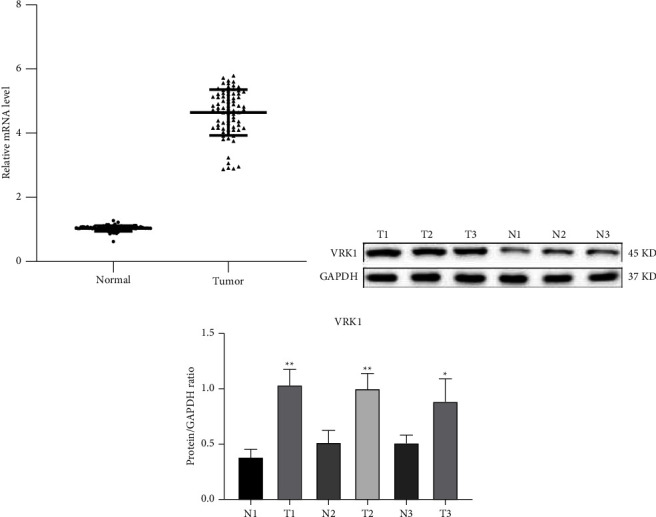
(a) PCR determination of VRK1 mRNA expression levels in cancer tissues of 25 clinical pairs of LUSC patients and their paraneoplastic normal tissues; (b) WB detection of VRK1 protein expression in three pairs of cancer and paraneoplastic tissues; (c) quantitative plots.

The postoperative LUSC tissues and paracancerous tissues of 25 LUSC patients were collected for the detection of VRK1 mRNA expression, and the study showed that the VRK1 gene expression level in cancer tissues was higher than that in paracancerous tissues (Figure 2(a)). In addition, the expression levels of VRK1 protein in three pairs of lung cancer and paraneoplastic tissues were examined by WB assay, and the results showed that the protein expression levels of VRK1 in lung cancer tissues were significantly higher than those in paraneoplastic tissues (*P* < 0.05) (Figures 2(b) and 2(c)).

### 3.3. Downregulation of VRK1 in NCI-H520 and SK-MES-1

**Figure 3:**
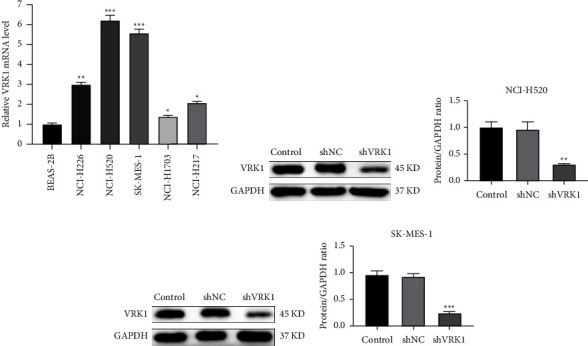
(a) VRK1 mRNA levels in the BEAS-2B cell line and five LUSC cell lines; (b) WB validation of NCI-H520 knockdown VRK1 efficiency and (c) quantification plots; (d) WB validation of SK-MES-1 knockdown VRK1 efficiency; and (e) quantification plots.

Five LUSC cell lines and BEAS-2B (human normal lung epithelial cells) were selected for the detection of VRK1 expression levels, and the results showed that VRK1 expression levels were significantly higher in lung cancer cell lines than in normal lung cell lines. Among them, VRK1 was expressed at the highest level in NCI-H520 and SK-MES-1, and these two cell lines were selected for subsequent experiments (Figure 3(a)). Then VRK1 was knocked down in NCI-H520 and SK-MES-1 by cell transfection, and WB results showed that VRK1 was successfully knocked down in both cell lines (Figures 3(b)–3(e)).

### 3.4. Effect of VRK1 Downregulation on LUSC Cell Proliferation

**Figure 4:**
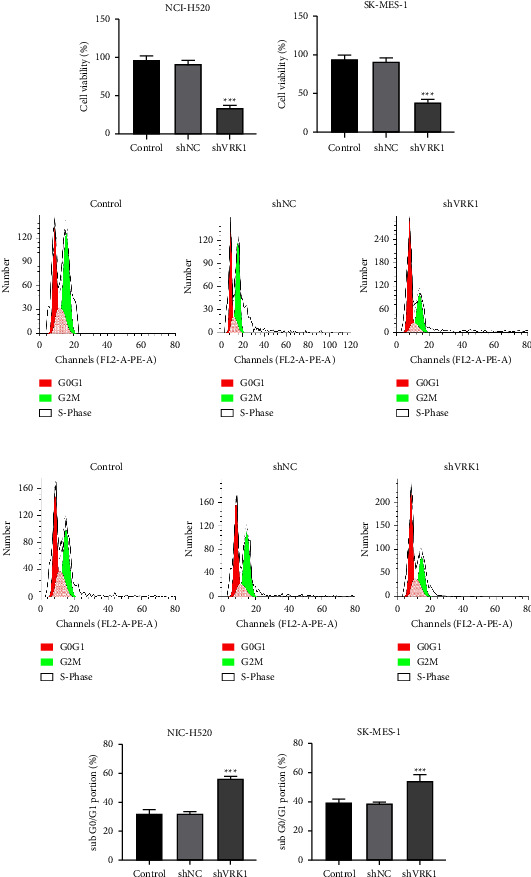
(a-b) CCK8 assay of cell activity in shVRK1-transfected, shNC-transfected, and control groups of NCI-H520 and SK-MES-1 cells; (c–d) flow cytometry assay of cell cycle in shVRK1-transfected, shNC-transfected, and control groups of NCI-H520 and SK-MES-1 cells and (e–f) their quantitative plots.

CCK8 results showed that the cell viability of the shVRK1-transfected group of NCI-H520 and SK-MES-1 was significantly lower than that of the shNC group (*P* < 0.001) (Figures 4(a) and 4(b)). To further investigate the effect of VRK1 on LUSC cell proliferation, we investigated the reason for the reduced activity of both cell lines. Flow cytometry assays showed that the cells in the *G*0/*G*1 phase of NCI-H520 and SK-MES-1 in the shVRK1 group were significantly more (*P* < 0.001) than those in the shNC group (Figures 4(c)–4(f)), and cell cycle arrest resulted in inhibition of LUSC cell proliferation.

### 3.5. Effect of VRK1 Downregulation on LUSC Cell Migration

**Figure 5:**
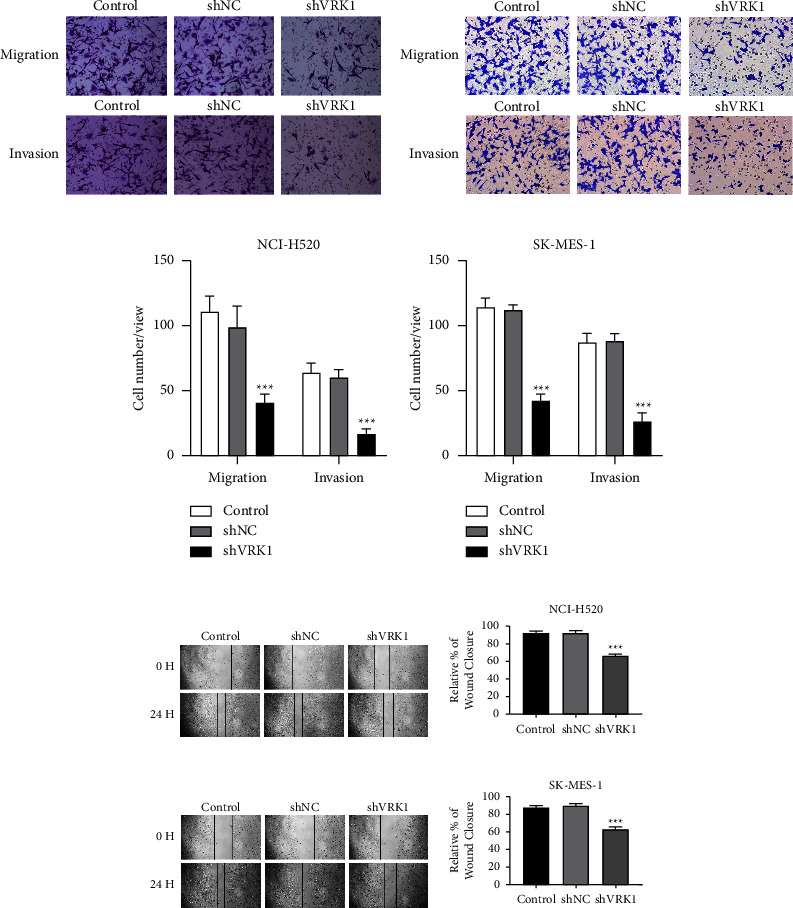
(a, b) Transwell assay to detect cell migration and invasion ability of the shVRK1-transfected group, the shNC-transfected group, and the control group of NCI-H520 and SK-MES-1 cells and their (c, d) quantitative plots; (e) cell scratch assay to detect cell migration ability of the shVRK1-transfected group, the shNC-transfected group, and the control group of NCI-H520 cells and their (f) quantitative plots; (g) cell scratch assay to detect cell migration ability of the shVRK1 transfected group, the shNC transfected group, and the control group of SK-MES-1 cells and their (h) quantitative plots.

The results of the wound healing assay and Transwell assay showed that the healing rate and cell migration number of NCI-H520 and SK-MES-1 cells were significantly lower in the shVRK1 group compared to the shNC group (*P* < 0.001) (Figure 5). The effect of VRK1 downregulation on LUSC cell migration was verified.

### 3.6. Effect of VRK1 Downregulation on Apoptosis in LUSC Cells

**Figure 6:**
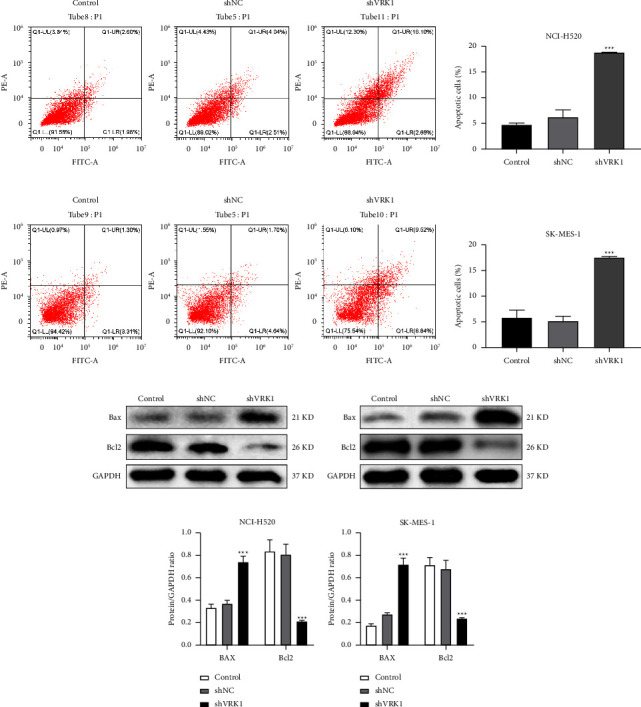
(a) Flow cytometry detection of apoptosis in shVRK1-transfected, shNC-transfected, and control groups of NCI-H520 cells and (b) their quantification; (c) flow cytometry detection of apoptosis in shVRK1-transfected, shNC-transfected, and control groups of SK-MES-1 cells and (d) their quantification; (e, f) WB assay of apoptosis index protein expression levels in shVRK1-transfected, shNC-transfected, and control groups of NCI-H520 and SK-MES-1 cells and (g, h) their quantification.

Flow cytometry analysis showed that the apoptosis rate of both NCI-H520 and SK-MES-1 cells was significantly increased after the knockdown of VRK1 compared to the shNC group (*P* < 0.001) (Figures 6(a)–6(d)). To further verify the effect of VRK1 downregulation on apoptosis in LUSC cells, western blot experiments were performed, and it was found that the expression level of BAX was significantly higher in NCI-H520 cells than in the shNC group after the knockdown of VRK1, while the expression level of bcl2 was significantly lower than in the shNC group (Figures 6(e) and 6(f)), and the same was true for SK-MES-1 cells (Figures 6(g) and 6(h)).

### 3.7. Effect of VRK1 Downregulation on DDR in LUSC Cells

**Figure 7:**
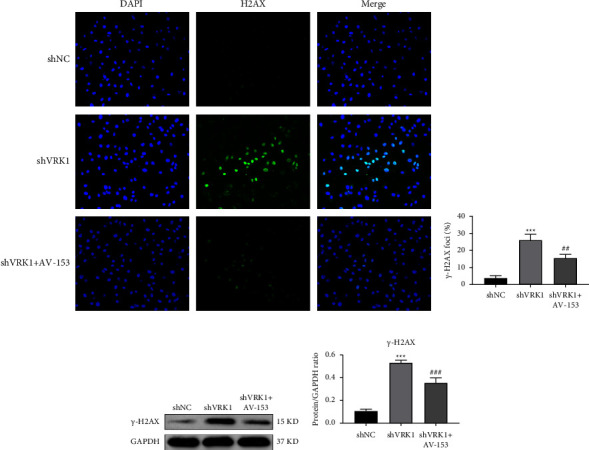
(a) Immunofluorescence assay to detect DNA damage in shVRK1-transfected group, shNC-transfected group, and control group of NCI-H520 cells and (b) quantification plot of *γ*-H2AX expression level; (c) WB assay to detect *γ*-H2AX in shVRK1 transfected group, shNC transfected group and shVRK1 + AV-153 group of NCI-H520 cells by -H2AX protein expression levels and their (d) quantified plots.

As shown by the immunofluorescence assay, the extent of DDR in NCI-H520 cells was significantly increased after the knockdown of VRK1 compared with shNC (*P* < 0.001), while the DNA of NCI-H520 cells was significantly restored after the addition of AV-153 (DNA damage repair agent) (*P* < 0.01) (Figures 7(a) and 7(b)). In addition, western blot experiments further verified the effect of VRK1 downregulation on DDR of LUSC cells, and the results showed that the *γ*-H2A.X expression level of NCI-H520 cells was significantly increased after knocking down VRK1 compared with the shNC group, while the *γ*-H2A.X expression level of NCI-H520 cells was significantly increased after adding AV-153 compared with the shVRK1 group, which was significantly reduced (Figures 7(c) and 7(d)).

### 3.8. Effect of VRK1 Downregulation on LUSC Tumors in Mice

**Figure 8:**
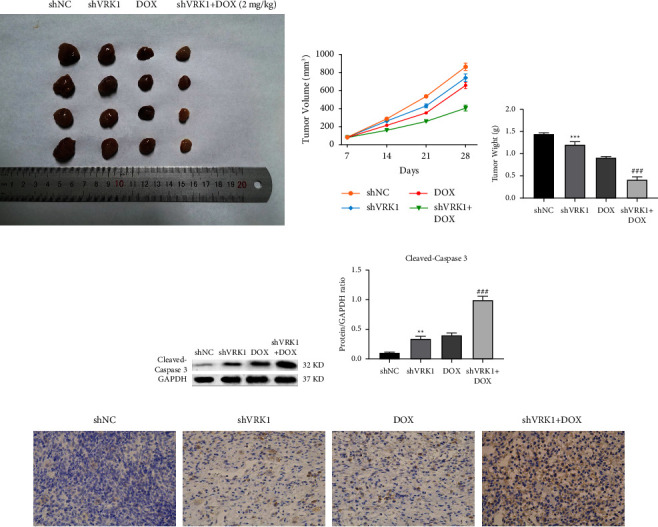
(a) Pictures of LUSC tumors in shVRK1-transfected, shNC-transfected DOX and shVRK1 + DOX groups of mice at 28 days and their (b) volume change graphs; (c) LUSC tumor weights in shVRK1-transfected, shNC-transfected DOX, and shVRK1 + DOX groups of mice at 28 days; (d) apoptosis of LUSC tumor cells in shVRK1-transfected, shNC-transfected DOX, and shVRK1 + DOX groups of mice at 28 days and (e) their quantification graphs with (f) microscopic observation.

Within 28 days, the tumor volume growth rate was the fastest in the shNC group, which was slowed down after knocking down VRK1, and the lung cancer tumor volume growth rate was the slowest in the shVRK1 + DOX group (Figures 8(a) and 8(b)). And the tumor weight of lung cancer was significantly lower after knockdown of VRK1 compared with shNC, while the tumor weight of the shVRK1 + DOX group was significantly lower compared with the shVRK1 group (Figure 8(c)). In addition, the caspase3 assay further verified the effect of VRK1 downregulation on mouse LUSC tumors, and the results showed that the tumor apoptosis level was significantly increased in the shVRK1 group compared with the shNA group (*P* < 0.01), while the tumor apoptosis level was further significantly decreased in the shVRK1 + DOX group compared with the shVRK1 group (*P* < 0.001) (Figures 8(d) and 8(f)).

## 4. Discussion

Lung cancer kills 1,589,000 people worldwide each year [11]. Advanced squamous lung cancer has very limited treatment options and a poor overall prognosis due to the lack of effective targeted drugs [12]. In this study, we found that VRK1 was highly expressed in LUSC and confirmed the effect of VRK1 downregulation on the migratory ability of LUSC cells at the cellular level. VRK1 downregulation through DDR could inhibit cell proliferation and block cells more in the G0/G1 phase. And in vivo experiments showed that downregulation of VRK1 inhibited tumor growth and promoted tumor cell apoptosis, and the effect was enhanced after combining with DOX, which showed a strong antitumor effect with certain clinical significance.

Chemotherapy is currently the main treatment for advanced squamous lung cancer, but the clinical efficacy has been at a plateau for a long time [13]. Therefore, researchers have gradually shifted their research focus to immunotherapy. Among them, the therapeutic effect of gene immunization on LUSC has gradually become a hot topic of research, although the effect of VRK1 on the migration and invasion of gastric cancer cells has been demonstrated [14]. However, the mechanism of action between VRK1 and LUSC remains to be investigated. VRK1 has been associated with poor prognosis in several types of tumors [15]. It has been previously shown that VRK1 translation can be regulated, thereby promoting lung cancer cell proliferation [16]. Similar conclusions were reached in the present experiment, where we found that VRK1 expression levels were significantly higher in lung cancer tumor tissues than in normal tissues, as well as in the relevant assays of clinical data. This shows that the expression of VRK1 is closely related to the development of LUSC.NCI-H520 and SK-MES-1 are two common LUSC cell lines, through which Na et al. found that the knockdown of GNA13 could inhibit LUSC [17]. In the present study, it was also found that VRK1 expression was highest in both NCI-H520 and SK-MES-1 cell lines, and the cell viability of NCI-H520 and SK-MES-1 in the shVRK1 group was significantly lower than that in the shNC group, and SK-MES-1 in the shVRK1 group had significantly lower cell viability than in the shNC group. Previously, it was shown that VRK1 expression could promote cell proliferation in myeloma by regulating cell cycle-related proteins [18]. The current study found that the inhibition of LUSC cell proliferation was due to depletion of VRK1, which induces the cell cycle arrest in the G0/G1 phase, which is also consistent with Carrion-Marchante et al. [19]. VRK1 promotes cell proliferation and migration in gastric cancer cells [14], esophageal squamous cell carcinoma [20], bladder cancer [21], and we derived the same results in LUSC. This suggests that the downregulation of VRK1 has an inhibitory effect on cell proliferation and migration in lung squamous carcinoma.

The B lymphocytoma-2 gene (bcl-2) and Bcl-2-associated X protein (BAX) are important indicators of apoptosis. In this experiment, the apoptosis rate of LUSC cells in the shVRK1 group was also found to be significantly higher than that in the shNC group by apoptosis indicators. This suggests that knockdown of VRK1 may lead to an increased level of apoptosis in LUSC, and it has also been previously shown that VRK1 can promote apoptosis in breast cancer cells [22] and posttransplant cardiomyocytes [23].

Lung cancer is a complex disease caused by a combination of environmental and genetic factors, of which DDR is an important component [24]. *γ*H2AX is formed by phosphorylation of H2AX in the conserved region at the C-terminus of serine-139 after DNA double-strand break, and histone H2AX plays an important role in DDR repair, cell cycle checkpoint regulation, maintenance of genomic stability, and tumor suppression [25]. And the DNA repair agent AV-153 plays an important role in DNA repair [26]. It has been shown that overexpression of BTG2 in lung cancer cells leads to an increase in the number of DDR-induced*γ*-H2AX lesions [27]. However, the effect of VRK1 on DDR has yet to be studied. The present experiment revealed that shRNA-mediated VRK1 deletion in the NCI-H520 cell line produced *γ*-H2A.X. This suggests that VRK1 downregulation induces DNA double-strand breaks (DSBs) by interfering with the repair system; thus, we predict that VRK1 inhibitors may lead to the accumulation of DNA damage by blocking the function of VRK1 in DDR.

Doxorubicin (DOX) is a cycle nonspecific drug that inhibits DNA and RNA synthesis and is an effective and inexpensive broad-spectrum antitumor drug [28]. DOX has previously been shown to have synergistic anticancer effects with both epalrestat [29] and l-arginine [30]. In the present study, we found that both VRK1 downregulation and DOX administration inhibited tumor growth, and the combined effect of VRK1 downregulation and DOX on promoting LUSC apoptosis was greater than that of DOX alone.

In summary, VRK1 exhibited strong antitumor effects with certain clinical significance, providing a new theoretical basis for the pathogenesis and prognostic assessment of LUSC as well as a new strategy for molecularly targeted therapy of LUSC. However, only two cell lines were selected for this experiment, and further investigation of the mechanism of VRK1 action on LUSC requires assays on a larger range of LUSC cells. Further demonstration of the DDR effects of VRK1 downregulation is needed in controlled trials with additional types of DDR repair drugs. The current treatment methods for tumors are in urgent need of innovation, and targeted therapy as a new approach still has many shortcomings and needs more research and exploration.

## Figures and Tables

**Table 1:** Clinicopathologic characteristics of 25 LUSC patients.

Parameter		Total (*n* = 25)
Gender	Male	11 (44%)
	Female	14 (66%)

Age (years)	<60	23 (92%)
	≥60	2 (8%)

Tumor size (cm)	<3	20 (80%)
	≥3	5 (20%)

Tumor location	Left	15 (60%)
	Right	10 (40%)

Histological grade	Middle or low	20 (80%)
	High	5 (20%)

Smoking history	Yes	21 (84%)
	No	4 (16%)

Staging	I	8 (32%)
	II	11 (44%)
	III	6 (24%)

## Data Availability

The datasets generated for this study are available upon request to the corresponding author.
